# Supplementary figures and images for: Electric pulse exposure reduces AAV8 dosage required to transduce HepG2 cells
Source: PLoS One. 2024 Apr 30;19(4):e0298866. doi: 10.1371/journal.pone.0298866 (PMC11060518; doi:10.1371/journal.pone.0298866)

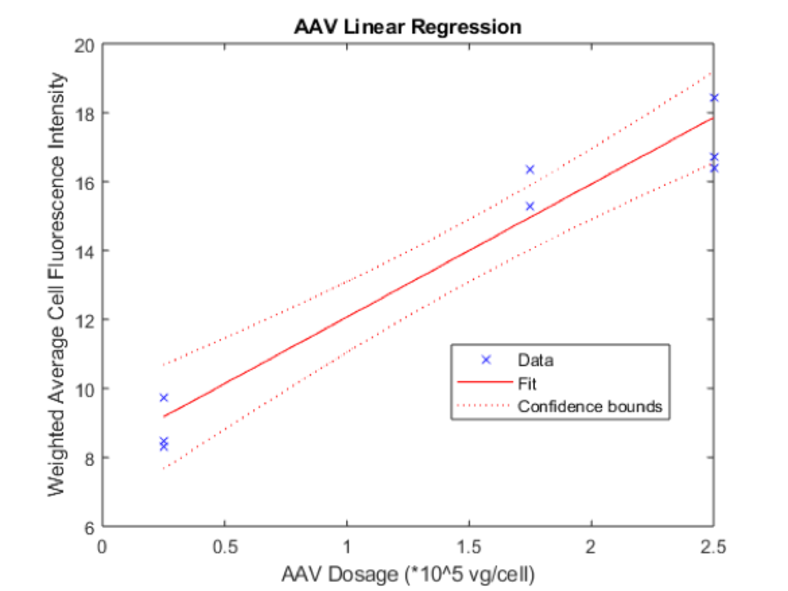

Supplement: S1 Fig — To substantiate the conclusions drawn in our main text regarding the impact of EP on transduction efficiency, we conducted a set of controlled experiments. The objective was to quantitatively establish the relationship between AAV dosage and the resultant transduction efficiency within HepG2. The experiments involved the application of three distinct dosages of AAV: 0.25×10^5^, 1.75×10^5^, and 2.5×10^5^ vg/cell. Post-transduction, the cells were imaged and analyzed to obtain fluorescence intensity measurements, which served as a proxy for transduction efficiency. These intensity values were then normalized and rescaled to create a standard range from 0 to 100, facilitating comparison across varying AAV dosages. A linear regression analysis was performed on the weighted average fluorescence intensities against the corresponding AAV dosages. This analysis yielded an R-squared value of 0.927, indicating a linear relationship between AAV dosage and transduction efficiency. (TIF) [file pone.0298866.s002.tif]
